# Supplementary material for: Does lack of parental involvement affect school dropout among Indian adolescents? evidence from a panel study
Source: PLoS One. 2021 May 10;16(5):e0251520. doi: 10.1371/journal.pone.0251520 (PMC8109829; doi:10.1371/journal.pone.0251520)
Supplement: Attachment — Submitted filename: plos review.docx [file pone.0251520.s001.docx]

The study fills a gap in the current knowledge space. It will add to the existing database and inform policy makers. However, in order to make it more robust, the following are suggested:

There could be certain concerns about the cohort size, as the adolescents were only about 50% of the original children covered. Although that seems to have been addressed, some more explanation of whether that skews the results in any manner can be made.

Another issue worth flagging is that in India, a child completes class ten by the age of 16 years, or 17 at most. Thus, there is a natural drop out as it were, around that age. If a larger number of children in the cohort are grouped towards the 17-18 years band, they would in any case drop out; either for work, or lack of higher secondary schools or colleges nearby. This could skew the results, and needs to be addressed. Besides, distance is a major factor in parents not sending their children for higher education. It will add to the study if this can be addressed.

The Right to Education Act in India addresses the entitlements of children up to 14 years. The authors could analyze whether this is a factor that comes into play in dropouts, as many benefits associated with the Act are not available after that age.

The study has brought out the need for parental involvement in furthering the educational attainment of their children. Towards this end, some policy level suggestions can be incorporated. These could be structured parent teacher meetings, some activities for the family as a part of the “home work” etc. Even though the dropout rates from public schools are higher as brought out by the study, yet these recommendations/inputs, given in a structured manner, can help these schools have a greater stakeholder involvement, and create accountability for the teachers there.
